# Two Toxic Lipid Aldehydes, 4-hydroxy-2-hexenal (4-HHE) and 4-hydroxy-2-nonenal (4-HNE), Accumulate in Patients with Chronic Kidney Disease

**DOI:** 10.3390/toxins12090567

**Published:** 2020-09-03

**Authors:** Christophe O. Soulage, Caroline C. Pelletier, Nans Florens, Sandrine Lemoine, Laurence Dubourg, Laurent Juillard, Fitsum Guebre-Egziabher

**Affiliations:** 1 CarMeN Laboratory, INSERM U1060, INRA, Université Claude Bernard Lyon 1, F-69500 Bron, France; caroline.pelletier02@chu-lyon.fr (C.C.P.); nans.florens@chu-lyon.fr (N.F.); sandrine.lemoine01@chu-lyon.fr (S.L.); laurent.juillard@univ-lyon1.fr (L.J.); fitsum.guebre-egziabher@chu-lyon.fr (F.G.-E.); 2 Hospices Civils de Lyon, Department of Nephrology–Hypertension-Dialysis, Hôpital E. Herriot, F-69003 Lyon, France; laurence.dubourg@chu-lyon.fr; 3 Faculté de Médecine, Université Claude Bernard Lyon-1, Lyon Est, F-69003 Lyon, France

**Keywords:** lipid peroxidation, lipid aldehydes, lipid peroxidation by-products, renal disease, oxidative stress, omega 3 fatty acids, kidney, glomerular filtration rate, polyunsaturated fatty acids, uremic toxins

## Abstract

Lipid aldehydes originating from the peroxidation of n-3 and n-6 polyunsaturated fatty acids are increased in hemodialysis (HD) patients, a process already known to promote oxidative stress. However, data are lacking for patients with chronic kidney disease (CKD) before the initiation of HD. We prospectively evaluated the changes of plasma concentrations of two major lipid aldehydes, 4-HHE and 4-HNE, according to the decrease of glomerular filtration rate (GFR) in 40 CKD and 13 non-CKD participants. GFR was measured by inulin or iohexol clearance. Thus, 4-hydroxy-2-nonenal (4-HNE) and 4-hydroxy-2-hexenal (4-HHE) were quantitated in plasma by gas chromatography coupled with mass spectrometry and their covalent adducts on proteins were quantified by immunoblotting. On the one hand, 4-HHE plasma concentration increased from CKD stage I–II to CKD stage IV–V compared to non-CKD patients (4.5-fold higher in CKD IV–V, *p* < 0.005). On the other hand, 4-HNE concentration only increased in CKD stage IV–V patients (6.2-fold, *p* < 0.005). The amount of covalent adducts of 4-HHE on plasma protein was 9.5-fold higher in CKD patients than in controls (*p* < 0.005), while no difference was observed for 4-HNE protein adducts. Plasma concentrations of 4-HNE and 4-HHE are increased in CKD IV–V patients before the initiation of hemodialysis.

## 1. Introduction

Patients with chronic kidney disease (CKD) exhibit a high incidence rate of cardiovascular diseases [1,2]. Oxidative stress, i.e., an imbalance between production of reactive oxygen and nitrogen species and antioxidant systems, is a hallmark of the uremic syndrome [3,4,5]. Oxidative stress further promotes the inflammatory process, accelerates renal injury, and favors cardiovascular dysfunctions [6,7,8,9]. During this process, cell and tissue damages can result from a direct attack by radical species, but also from several oxidation by-products resulting from the oxidative breakdown of biomolecules. In contrast to the radical species, these secondary oxidation by-products can diffuse within the body fluids to propagate the deleterious effects of oxidative stress in remote tissues from their production site. Polyunsaturated fatty acids (PUFAs) are major targets of oxidative stress, and their oxidation generates numerous toxic electrophilic compounds [10]. Several oxidation by-products are generated from fatty acid peroxidation, a non-enzymatic process initiated by a free radical attack on the double bonds of PUFAs. Lipid peroxidation triggers the production of many reactive carbonyl compounds (RCCs) such as malondialdehyde (MDA), acrolein or 4-hydroxy-alkenals, among which the more studied are 4-hydroxy-2-nonenal (4-HNE) and 4-hydroxy-2-hexenal (4-HHE) [10]. In particular, 4-HNE is a nine-carbon lipid aldehyde issued from the peroxidation of n-6 PUFAs [11], while 4-HHE is a six-carbon lipid aldehyde issued from the peroxidation of n-3 PUFAs [12]. Because of their reactivity, the lipid aldehydes were proved to be involved in many pathological processes [13]. Moreover, 4-HNE and 4-HHE exhibit important electrophilic properties that make them prone to react with numerous classes of biomolecules such as lipids, nucleic acids or proteins (to form advanced lipoperoxidation by-products) [13,14,15]. The toxicity of lipid aldehydes is mainly due to their potency to covalently modify proteins. Indeed, HNE reacts with sulfhydryl and amino groups from cysteine and histidine residues [16,17]. Protein adducts have been detected in many circulating proteins including serum albumin [18] and various class of lipoproteins (LDL, HDL) [19,20]. While 4-HNE received extensive attention, the toxicity of 4-HHE is however less well documented [21]. We have previously demonstrated that the cytoxicity of 4-HHE and 4-HNE was related with their ability to form covalent adducts on proteins [22] that can significantly damage proteins. For instance, several enzymes are inactivated following structural modification by lipid aldehydes [23,24,25,26]. Most RCCs identified in the plasma of CKD patients are derived from carbohydrates, such as glyoxal, methylglyoxal or pentosidine [27,28]. In contrast, RCCs from lipid origin only received limited attention in CKD. Only one study reported that the concentrations of other lipid aldehydes (length 6–12 carbons) were increased in HD patients [29]. In spite of this limited set of data, 4-HNE and 4-HHE were recognized in 2013 as uremic toxins, belonging to the group of water-soluble/low molecular weight molecules, by the European Uremic Toxin Workgroup (EuTox, http://www.uremic-toxins.org/) [30]. Renal replacement therapy (RRT) such as hemodialysis is known to increase oxidative stress [31] and thus promote lipid peroxidation. However, there is no data in the literature focusing on the plasma levels of reactive lipid aldehydes in non-dialysed CKD patients. The aim of the present study was to quantify the concentrations of two major lipid aldehydes, 4-HNE and 4-HHE, and to explore their relationship with the decline in glomerular filtration rate (GFR) in CKD pre-dialysis patients.

## 2. Results

### 2.1. Patient Data

Forty patients with CKD stage 1 to end-stage 5 (aged 47 ± 14 years) and 13 non-CKD participants (aged 44 ± 13 years, *p* = 0.235) were included in the present study. The etiology of renal diseases was nine glomerulonephritis (but none from diabetes), six renovascular diseases (including three nephroangiosclerosis), six solitary kidney, four tubulointerstitial diseases, five cystic kidney disease, and 10 unknown etiologies. Out of the 40 CKD patients, 14 were on antihypertensive drugs (35%, *p* = 0.045) while eight were on lipid lowering drugs (20%, *p* = 0.322). The main characteristics of the CKD patients and controls are summarized in Table 1. As expected, mGFR (*p* < 0.001), plasma creatinine (*p* < 0.001) and urea (*p* < 0.001) were the main differences between groups.

### 2.2. Plasma Concentration of 4-hydroxy-alkenals Increases in Patients with Severe Loss of Kidney Function

**Figure 1:**
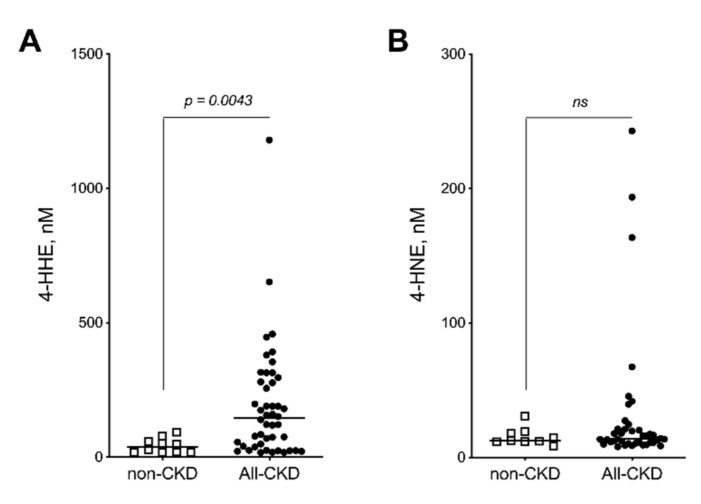
Plasma free concentration of 4-HHE and 4-HNE are increased in CKD patients. Concentrations of 4-HHE (**A**) and 4-HNE **(B**) were quantified in plasma from non-CKD subjects (*n* = 13) and chronic kidney disease (CKD stage 1 to 5, *n* = 40) patients by GC-MS as described in methods. The horizontal bar indicates the median. *p* < 0.05 was considered significant.

The measurement of 4-HHE and 4-HNE in plasma from CKD and non-CKD subjects was performed using gas chromatography–mass spectrometry (GC-MS) [32,33] (Figure 1).

**Figure 2:**
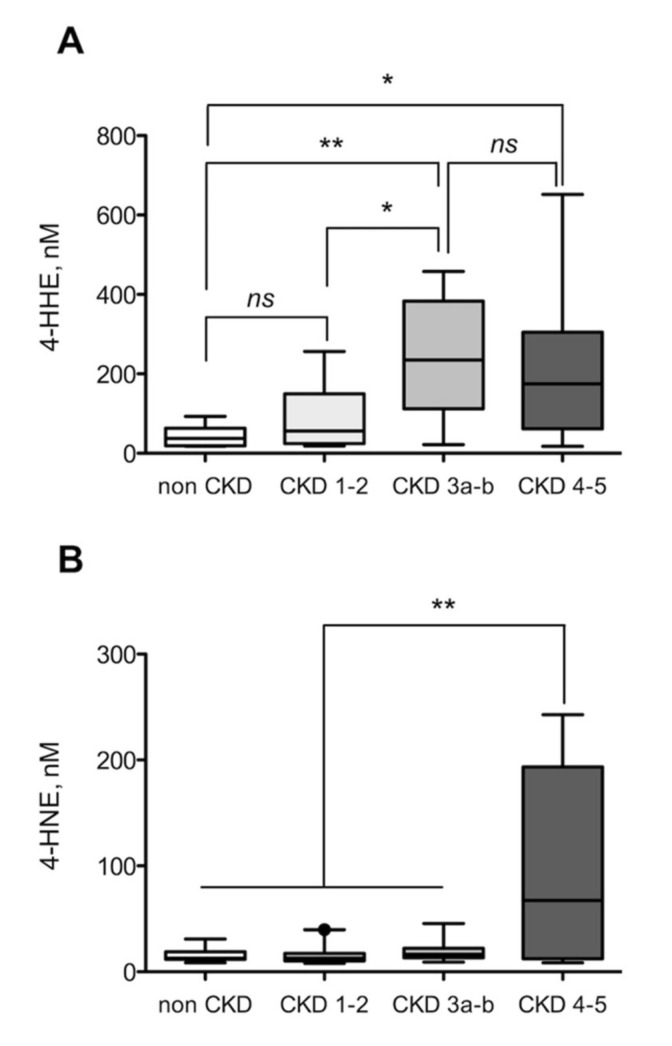
Plasma free concentrations of 4-HNE and 4-HHE are increased in CKD patients according to the stage of chronic kidney disease. 4-HHE (**A**) and 4-HNE (**B**) concentrations were measured in plasma from non-CKD subjects (*n* = 13) and chronic kidney disease (CKD stage 1 to 5, *n* = 40) patients by GC-MS as described in methods. On the boxplots, the median is indicated by a horizontal bar, the interquartile range as a box and the 5th to 95th percentile as the “whiskers”. GFR was measured using inulin or iohexol clearance. Data are presented as median (interquartile range). * *p* < 0.05, ** *p* < 0.01, ns non-significant.

Plasma 4-HHE concentration was 37 (interquartile range (IQR): 17–63) nmol/L in non-CKD subjects while it was 145 (40–284) nmol/L in CKD patients (3.9-fold, Mann–Whitney U test, *p* < 0.005) (Figure 1A). Surprisingly, no significant difference was noticed in the concentrations of 4-HNE (non-CKD vs CKD, 13 (12–19) nmol/L vs 14 (11–21) nmol/L, Mann–Whitney U test, *p* = 0.679) (Figure 1B). Plasma 4-HHE concentration gradually increased from CKD stage 1–2 to CKD stage 4–5 when compared to non-CKD subjects (Figure 2A). However, this increase was statistically significant from CKD stage 3 (*p* < 0.01 vs control, *p* < 0.05 vs CKD stage 1–2) and was 4.5-fold higher in CKD 4–5 patients (174.6 (61.5–305.2) nmol/L) compared with non-CKD subjects (37.3 (18.6–63.0) nmol/L, Dunn’s test, *p* < 0.005). In contrast to 4-HHE, 4-HNE concentrations did not gradually rise from CKD stage 1–2 to CKD stage 4–5 (Figure 2B). Indeed, concentrations were only significantly increased for CKD stage 4–5 patients and were 6.2–fold higher (67.4 (8.6–97.8) nmol/L) compared with non-CKD subjects (12.7 (11.8–18.8) nmol/L, Dunn’s test, *p* < 0.005).

### 2.3. Advanced Lipoperoxidation By-Products

**Figure 3:**
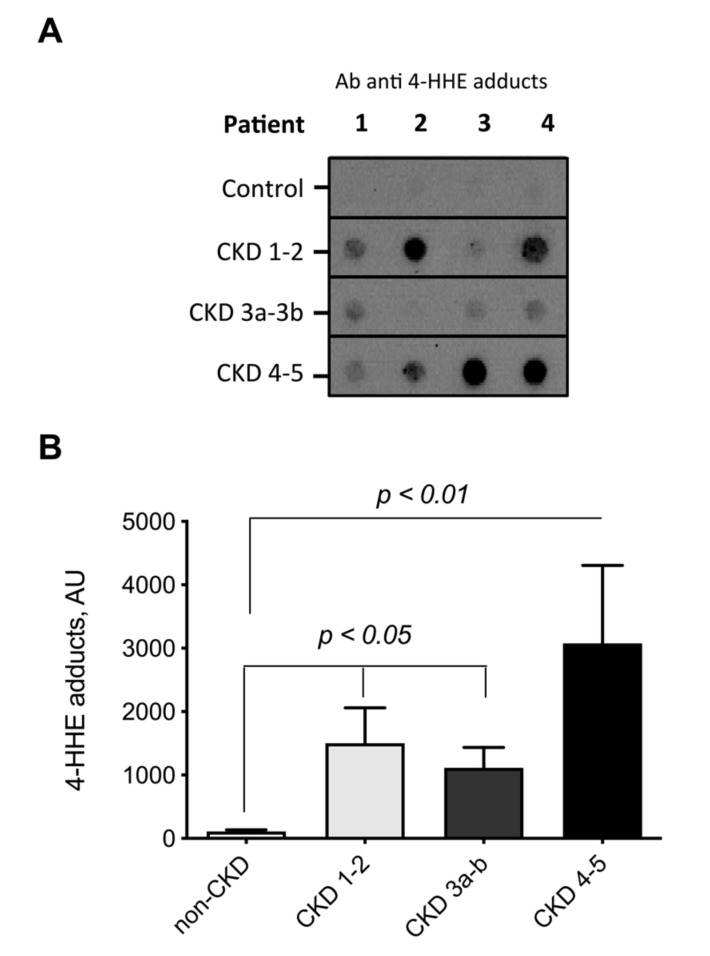
Measurement of 4-HHE Michael adduct on plasma proteins from non-CKD subjects and CKD patients. Michael adducts of 4-HHE were detected using specific antibodies directed against 4-HHE protein adducts (dot-blot). (**A**) Typical blots. (**B**) Quantification data, obtained by densitometry, are presented as median (interquartile range). Differences were considered significant at the *p* < 0.05 level. Note that 4-HHE adducts on proteins increased with the decline of renal function.

**Figure 4:**
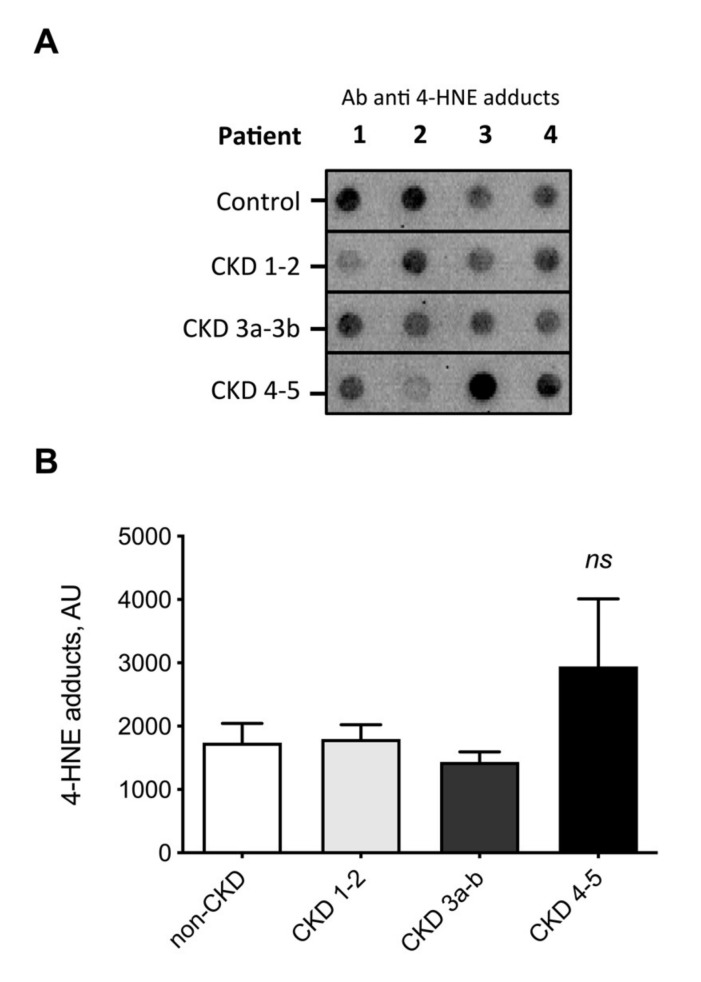
Measurement of 4-HNE Michael adduct on plasma proteins from non-CKD subjects and CKD patients. Michael adducts of 4-HNE were detected using specific antibodies directed against 4-HNE protein adducts (dot-blot). (**A**) Typical blots. (**B**) Quantification data, obtained by densitometry, are presented as median (interquartile range). No difference was found to be significant at the *p* < 0.05 level.

It is known that 4-HNE and 4-HHE are prone to react with proteins to form covalent adducts [10]. We therefore used specific antibodies directed against Michael adducts of 4-HNE and 4-HHE to detect covalent adducts on plasma proteins using immunoblotting (i.e., dot blot). Regardless of CKD stage, CKD patients exhibited an increased level of 4-HHE adducts on plasma proteins (Figure 3A,B). The level of 4-HHE protein adducts was 115 (92–147) arbitrary unit (AU) in non-CKD subjects and 1093 (587–3984) AU in patients with CKD (Mann–Whitney U test, *p* < 0.005), i.e., a 9.5-fold increase. Surprisingly, no difference was noticed in 4-HNE protein adducts (1617 (1129–2501) and 1863 (1450–2524) AU for non-CKD and CKD patients, respectively (Figure 4).

To obtain further insight, ALEs were studied according to the actual stage of CKD which was evaluated with measured GFR. 4-HHE protein adducts gradually increased with the decline of GFR (Figure 3B) while 4-HNE protein adducts were not correlated with the CKD stage (Figure 4A,B).

### 2.4. Correlation Study: Relationships between Plasma 4-hydroxy-alkenal Concentration and Clinical or Biochemical Parameters

The analyses of univariate correlations are presented in Table 2. Plasma 4-HHE concentration was positively correlated with age (Spearman r_s_ = 0.430, *p* < 0.005) and negatively with mGFR (Spearman r_s_ = −0.377, *p* < 0.05).

Plasma 4-HNE concentration negatively correlated with mGFR (Spearman r_s_ = −0.444, *p* < 0.005) and positively associated with age (Spearman r_s_ = 0.326, *p* < 0.05), plasma creatinine level (Spearman r_s_ = 0.628, *p* < 0.005) and urinary albumin/creatinine ratio (Spearman r_s_ = 0.307, *p* < 0.05). Plasma 4-HHE concentration positively associated with 4-HHE adducts on proteins (Spearman r_s_ = 0.661, *p* < 0.005) while no such trend was noticed for 4-HNE. As the plasma level of 4-HHE was significantly associated with age, we performed a multivariable linear model including demographic data (gender, age) and mGFR (Table 3). After adjustment for age and gender, mGFR remained a predictor of 4-HHE levels (β = −1.245, *p* < 0.05).

## 3. Discussion

Few studies previously reported that plasma lipid aldehydes (among which 4-HNE and 4-HHE) concentrations were increased in chronic hemodialysis patients [29]. However, no data is available in the literature regarding the actual concentration of 4-HNE and 4-HHE in CKD patients before initiation of renal replacement therapy, a process that exacerbates oxidative stress [31,34]. We observed that plasma concentrations of both 4-HNE and 4-HHE dramatically increase in CKD 4–5 patients. Plasma levels of 4-HHE (4.5-fold) and 4-HNE (six-fold) were higher in CKD 4–5 patients than in controls unambiguously evidencing the presence of oxidative stress before the initiation of dialysis. These results are in line with the report of Alhamdani et al. [29] who observed an increase in both 4-HHE (2.5-fold) and 4-HNE (seven-fold) in hemodialysis patients. These data suggest that oxidative stress could rather result from the uremic/inflammatory environment than from the renal replacement therapy (such as HD). Several works suggest that the retention of uremic toxins could contribute to increased oxidative stress in uremia [35,36,37,38,39,40,41,42].

In addition, 4-HHE and 4-HNE exhibited different patterns of accumulation. Indeed, while 4-HHE level gradually increases with the decline of GFR (reaching statistical significance at stage 3), 4-HNE only increased in CKD stage 4–5. Free 4-HHE concentration was positively correlated with the amount of 4-HHE adduct on plasma proteins while free 4-HNE concentration was not correlated with the 4-HNE adducts on plasma proteins (see Table 2). The relative proportion of 4-HHE and 4-HNE accumulated in end-stage renal disease (ESRD) deserves some comments. The proportion of 4-HHE to 4-HNE depends of two main factors: First, the availability of omega 3 PUFAs and omega-6 PUFAs, which largely depends from the dietary habits (e.g., intake of low/large amount of omega-3 PUFAs). Secondly, the relative activity of the various detoxifying enzymes and their respective affinity for 4-HNE and 4-HHE. While plasma level of omega-3 polyunsaturated fatty acids (PUFAs) is much lower than omega-6 PUFAs [43] omega-3 are however more prone to oxidation than omega-6 fatty acids. We recently measured plasma lipid aldehyde concentration in patients with type 2 diabetes [33] and performed simultaneously the assay of omega-3, omega-6 PUFAs (by GC coupled with flame ionization detector -FID) and their aldehyde by-products 4-HHE and 4-HNE (by GC-MS). Our data showed that, in T2D patients, one molecule of omega-3 PUFA out of 12,000 was oxidized into the form of 4-HHE while one molecule of omega 6 PUFA out 78,000 was oxidized into the form of 4-HNE. These data suggest a greater oxidizability of omega-3 PUFAs than omega-6 PUFAs. Detoxification of lipid aldehydes involves several enzymes, such as for instance glutathione S-transferase (GST), fatty aldehyde dehydrogenase (FALD) and/or aldehyde dehydrogenase (ALDH). Hubatsch et al. [44] reported that 4-HHE is a poor substrate for GSTA4-4 compared to HNE. Long et al. [45] demonstrated that HHE is a worse substrate than HNE for the mitochondrial ALDH5A while both of them are detoxified by aldose reductase [46]. Some differences in detoxification and disposition could therefore account for the different levels of 4-HNE and 4-HHE accumulated in ESRD patients. Some data suggest an overexpression of GST in hemodialysis patients [47] but there is no further information about the other detoxifying enzymes.

In an experimental model, increased lipid peroxidation was shown to play a pathophysiological role for glomerulonephritis [48]. A deleterious role of lipid peroxidation was also suggested in CKD [49]. Bae et al. (2011) [50] demonstrated in vitro that 4-HHE induces HK-2 tubular cells apoptosis, suggesting that it could contribute to pathogenesis of kidney injury. Moreover, 4-HHE treatment resulted in a decline of tubular cells viability associated with an over-expression of nuclear factor kappa-B (NF-κB) and a decrease of inhibitor of nuclear factor kappa-B kinase beta (IκBKβ). Nevertheless, the concentrations of 4-HHE used in this study (5–100 µM) were unrealistic, largely exceeding the concentrations found in human plasma from CKD patients [29] (i.e., <1µM, from the present study). However, the lipid peroxidation by-products play an important role in inflammation. 4-HNE and many other aldehydes displayed a chemotactic activity for leukocytes from a concentration of 0.1µM [51,52], suggesting that these compounds could contribute to micro-inflammation commonly observed in CKD.

Furthermore, 4-HHE and 4-HNE are reactive aldehydes that are prone to covalently bind to plasma proteins. Lipid aldehydes can react with proteins through the production of Schiff bases (involving the amino group of lysine residues) and/or Michael adducts (involving thiol or amino groups of cystein, lysine or histidine residues) [53]. This effect is therefore thought to be pivotal in the toxic effect of hydroxy-alkenals. Lipid aldehydes are able to covalently bind to serum albumin [18] and many other plasma proteins, and through change in their tridimensional structures, alter their binding capacity for other ligands. The lipid aldehydes exhibit therefore the double potency to behave as small molecules but also as protein bound uremic toxins. This type of interactions (i.e., covalent) with proteins is very different from more classical protein-bound retention solutes, such as indoxyl sulfate or *p*-cresyl sulfate. This covalent binding of lipid aldehydes can be regarded as irreversible in contrast to the other ligands, which display a competitive binding with two specific binding sites on albumin. It is worth noting that adducted proteins can actually exert deleterious biological activities [20,54] therefore behaving themselves as secondary toxins. In our study, in contrast to 4-HHE, the increase of plasma 4-HNE was not accompanied with a parallel increase of its protein adducts. This discrepancy may be related to the difference in the biological properties of the two aldehydes that is partially related to their carbon chain length (six and nine carbons for 4-HHE and 4-HNE, respectively). This structural difference may result in large difference of solubility and reactivity with amino acids. For instance, in vitro studies have demonstrated the formation of adducts incorporating multiple 4-HNE molecules [19]. Furthermore, as we only measured plasma concentrations, we cannot exclude a difference in tissue accumulation of 4-HNE adducts conferring intracellular toxicity. Indeed, 1%–8% of 4-HNE that is generated in cells would react with proteins and the majority of the target enzymes for 4-HNE are mitochondrial proteins [55]. Further studies are however needed to investigate the difference in metabolism of these two aldehydes in the context of CKD.

According to the European Uremic Toxin (EuTox) workgroup [30], a uremic toxin can be defined as a compound that (i) is excreted in urine and thus accumulates in patients with CKD as a result of the decline in renal clearance and (ii) that exerts deleterious effects on biological systems. 4-HHE and 4-HNE are partly excreted by the kidney in the form of 4-hydroxy-2-hexenoic acid (4-HHA), 4-hydroxy-2-nonenoic acid (4-HNA), or as mercapturic acid [56], but the kidney is not the major site of their metabolization. Indeed, the main site of hydroxyalkenals biotransformation is the liver [56] and the subsequent metabolites are excreted by the kidneys. Further, 4-HHE and 4-HNE do not accumulate in CKD as the sole result of a decreased renal clearance, and thus according to the EuTox definition, are not strictly speaking uremic toxins.

## 4. Conclusions

We reported in the present study that plasma levels of two lipids aldehydes, namely 4-HNE and 4-HHE, displayed increased level in CKD 4–5 patients. These data confirmed that the oxidative stress preexists in CKD prior to the initiation of renal replacement therapy (e.g., dialysis). Lipid aldehydes accumulated in ESRD as well as advanced lipoperoxidation end products (e.g., protein adducts) could, as has already been documented in many other diseases (e.g., diabetes), exerting a deleterious effect, and can therefore be regarded as a “uremic toxin like” compounds.

## 5. Materials and Methods

### 5.1. Chemicals

Unless otherwise specified, all solvents were from Carlo Erba (Peypin, France) and all chemicals from Sigma Aldrich (Saint Quentin Fallavier, France).

### 5.2. Ethic Statement

This research was approved by the local ethic committee (Comité de Protection des Personnes—Recherche Biomédicale, CPP Lyon Sud-Est IV under the reference DC-2012-1615, 2 July 2012) and conducted according to the ethical standards and the principles of the second Declaration of Helsinki. Prior to enrolment, all subjects involved in the present research had signed a written informed consent form.

### 5.3. Subjects

A total of 53 subjects (32 men), including 13 non-CKD subjects, were recruited from department of Nephrology of Edouard Herriot University Hospital (Lyon, France). This study was proposed to CKD patients referred for GFR measurement excluding renal transplant recipients. Exclusion criteria were age >65 years and the main pathologies other than CKD, known to be associated with oxidative stress (diabetes, obesity defined as a body mass index >30 kg/m^2^, systemic inflammatory, autoimmune diseases or known active cancer). Non-CKD patients were candidates for kidney donation.

### 5.4. Blood Sampling

After an overnight fast, blood samples were obtained by venipuncture and centrifuged at 3500× *g* for 10 min. Plasma was isolated, snap frozen in liquid nitrogen and stored at −80 °C until use.

### 5.5. Glomerular Filtration Rate Measurements (mGFR)

GFR was measured by inulin or iohexol clearances (expressed as mL/min per 1.73 m^2^). Urinary inulin clearance was performed for 32 subjects. After a priming dose, inulin (Polyfructosan [inutest], Laevosan, Linz, Austria) was perfused at constant flow for 3 h and urines were collected every 30 min by spontaneous voiding. Blood samples were obtained in the middle of each period of urine collection (3 to 4 collection periods of 30 min each). Inulin concentration was assayed by an enzymatic method. Inulin clearance was calculated using the following equation:

(1) $$GFR=\frac{U\times \;V}{P}$$

where U is urine concentration of inulin, V is urine volume and P is the plasma concentration of inulin. Plasma iohexol clearance was performed for 21 subjects. Iohexol (300 mg; Omnipaque; GE Healthcare SAS, Vélizy-Villacoublay, France) was injected intravenously and blood was drawn at 120, 180, and 240 min. The plasma concentration of iohexol was measured by HPLC. The GFR was calculated using the following equation:

(2) $$GFR=Slope\;\times \frac{Dose}{C_{0}}$$

where Slope refers to the slope of the plasma decay of iohexol concentration and C_0_ the plasma iohexol concentration at time 0 (corrected with the Bröchner–Mortensen equation). CKD stages were determined according to the K/DOQI (Kidney Disease Outcomes Quality Initiative) guidelines.

### 5.6. 4-hydroxy-2-alkenals Assay

**Figure A1:**
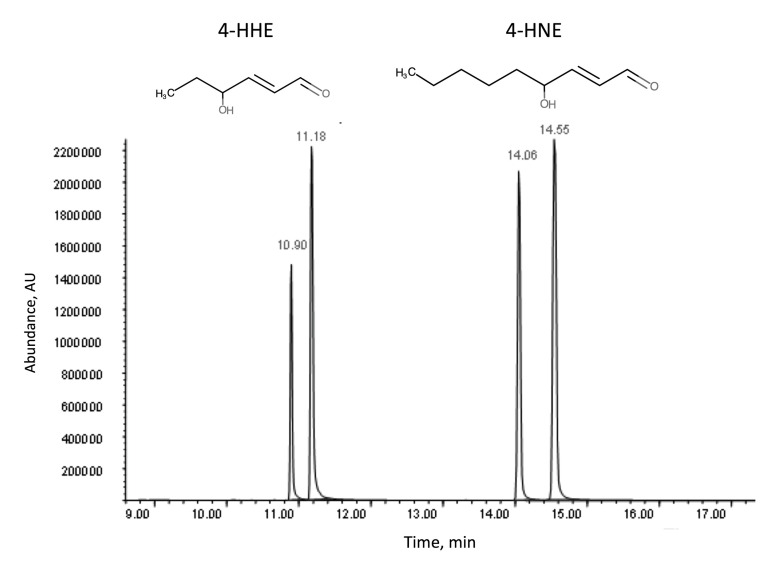
Typical chromatogram for 4-HHE and 4-HNE assay by GC-MS. NICI GC/MS reconstituted chromatogram of 4-HHE and 4-HNE derivatized as PFB oxime and TMS. Plasma sample was spiked with 4-HNE and 4-HHE. Ions at *m*/*z* 241, 271, 291 and 361 for 4-HHE and ions at *m*/*z*: 283, 313, 333 and 403 for 4-HNE, respectively. Two peaks are shown, corresponding to syn- and anti-isomers of derivatized 4-HHE and 4-HNE.

4-Hydroxy-2-alkenals were assayed by GC-MS as previously described [32,33]. Deuterated 4-HNE (4-HNE-CD_3_) and 4-HHE (4-HHE-CD_3_) were used as internal standards for quantification. Ions at *m*/*z* 283, 313, 333, 403 and 286, 316, 336 and 406 were measured for 4-HNE and 4-HNE-CD3, respectively. Ions at *m*/*z* 241, 271, 291, 361, and 244, 274, 294, 364 were measured for 4-HHE and 4-HHE-CD3, respectively (see a typical chromatogram in the Figure A1 in Appendix A).

### 5.7. Dot Blot: 4-HHE and 4-HNE Protein Adducts

Primary antibodies against HNE-Michael adducts (reference 393207) and against HHE-Michael adducts (reference NOF-N213730-EX) were from Calbiochem (San Diego, CA, USA) and Cosmobio (Tokyo, Japan), respectively. Further, 4-HHE and 4-HNE Michael adducts on plasma protein were evaluated by dot-blotting as previously described [22,33,57]. Briefly, 50 µg of proteins were loaded on a nitrocellulose membrane. Following saturation (using 5% bovine serum albumin), membranes were incubated overnight at 4 °C with primary antibodies. After incubation with HRP-coupled secondary antibodies (for 1h and 30 min at room temperature), membranes were analyzed for chemiluminescence (ECL plus, GE Healthcare) and quantitated by densitometry using Image J software (NIH, Bethesda, MD, USA, https://imagej.nih.gov).

### 5.8. Other Biochemical Measurements

Plasma urea, bicarbonate, hemoglobin, plasma proteins and serum albumin were measured by standard laboratory.

### 5.9. Statistical Analysis

Data were analysed using Graphpad Prism (GraphPad softwares, La Jolla, CA, USA) and R (https://www.r-project.org/) softwares. The data are expressed as mean ± 1 standard deviation (SD) or as median (interquartile range—IQR) when variables were not normally distributed. Distributions were tested for normality using Shappiro–Wilk test. Differences between groups were assessed by Kruskall–Wallis tests followed when appropriate by Dunn tests. Sex ratio and medications between groups were compared using Fisher exact test. Univariate analysis was performed using the Spearman rank correlation method. A *p* < 0.05 was considered as statistically significant in all analysis.

## Figures and Tables

**Table 1:** Demographic and clinical data for the non CKD and CKD participants.

	Non CKD			Stage 1–2			Stage 3a–3b			Stage 4–5			*p*-Value
Gender, M/F	9/4			8/11			4/6			9/2			0.092
Age, y	43.9	±	13.0	44.8	±	13.5	52.8	±	14.3	45.9	±	14.9	0.424
Body weight, kg	73.9	±	11.4	65.3	±	16.0	67.7	±	17.9	77.5	±	12.5	0.129
Height, m	1.72	±	0.10	1.72	±	0.12	1.67	±	0.10	1.75	±	0.12	0.285
BMI, kg/m^2^	24.9	±	2.1	23.0	±	2.7	23.9	±	4.0	25.2	±	3.0	0.158
Systolic BP, mmHg	130	±	14	127	±	20	135	±	18	136	±	18	0.487
Diastolic BP, mmHg	82	±	14	81	±	14	87	±	13	90	±	11	0.334
mGFR, mL/min/1.73m^2^	102	±	9	75	±	11	46	±	8	20	±	6	<0.001
Creatinine, µmol/L	66	±	17	82	±	17	127	±	41	393	±	149	<0.001
Urea, mmol/L	5.3	±	1.6	6.4	±	2.4	9.0	±	2.4	14.7	±	6.6	<0.001
Bicarbonate, mmol/L	26.3	±	2.0	25.2	±	2.9	24.6	±	2.7	22.6	±	2.9	0.143
Proteins, g/L	76.7	±	5.0	72.5	±	15.0	75.6	±	2.3	72.3	±	4.9	0.808
Hypertension, %	30.0			36.8			40.0			54.5			0.468
Lipid-lowering therapy, %	0			0			20			54.5			0.006
RAAS inhibitors, %	0			21.1			40			54.5			0.038

Data are expressed as mean ± SD. Abbreviations: BMI, body mass index, CKD, chronic kidney disease, BP, blood pressure, mGFR, measured glomerular filtration rate, RAAS, Renin-angiotensin-aldosterone system. GFR was measured using inulin or iohexol clearance as described in Methods. Differences were considered significant at the *p* < 0.05 level.

**Table 2:** Univariate correlations with 4-HNE or 4-HHE plasma concentrations.

	4-HNE		4-HHE	
	r_s_	*p*-Value	r_s_	*p*-Value
Age, y	0.326	0.022	0.430	0.004
BMI, kg/m^2^	0.068	0.640	0.253	0.098
MAP, mm Hg	0.161	0.269	0.223	0.145
Proteins, g.L^−1^	0.011	0.953	−0.201	0.305
4-HHE, ng/mL	0.146	0.318	-	-
Bicarbonate, mmol/L	−0.075	0.688	0.070	0.729
mGFR, mL/min/1.73m^2^	−0.444	0.002	−0.377	0.012
Urea, mmol/L	0.146	0.337	0.164	0.312
Creatinine, µmol/L	0.628	<0.001	0.152	0.326
Urine albumin/Creatinine ratio	0.307	0.042	−0.067	0.685
4-HNE protein adducts, AU	0.163	0.457	0.408	0.083
4-HHE protein adducts, AU	−0.043	0.858	0.661	0.003

Abbreviations: AU, arbitrary unit, BMI, Body mass index, 4-HHE, 4-hydroxy-2-hexenal, 4-HNE, 4-hydroxy-2-nonenal, mGFR: measured glomerular filtration rate, MAP, mean arterial pressure, mGFR was measured using inulin or iohexol clearance. Correlations were considered significant at the *p* < 0.05 level.

**Table 3:** Multivariable linear model showing association with 4-HHE plasma concentration.

	β-Coefficient	95% CI	*p*-Value
Outcome Variable: 4-HHE Plasma Concentration			
Predictor variables:			
Age, y	3.455	[0.825, 6.085]	0.012
Gender	11.66	[−57.73, 81.04]	0.735
mGFR, mL/min/1.73m^2^	−1.245	[−2.484, −0.006]	0.049
Intercept	44.8	[−115.8, 205.3]	0.575

The model was adjusted for demographic parameters (adjusted *r^2^* = 0.212, *p* = 0.009). Abbreviations: 4-HHE, 4-hydroxy-2-hexenal, 4-HNE, 4-hydroxy-2-nonenal, mGFR was measured using inulin or iohexol clearance. Gender was coded as 0 for female and 1 for male. Difference were considered significant at the *p* < 0.05 level.
